# Spatial Fluctuations in Expression of the Heterocyst Differentiation Regulatory Gene *hetR* in *Anabaena* Filaments

**DOI:** 10.1371/journal.pgen.1005031

**Published:** 2015-04-01

**Authors:** Laura Corrales-Guerrero, Asaf Tal, Rinat Arbel-Goren, Vicente Mariscal, Enrique Flores, Antonia Herrero, Joel Stavans

**Affiliations:** 1 Instituto de Bioquímica Vegetal y Fotosíntesis, CSIC and Universidad de Sevilla, Sevilla, Spain; 2 Department of Physics of Complex Systems, Weizmann Institute of Science, Rehovot, Israel; Max Planck Institute for Terrestrial Microbiology, GERMANY

## Abstract

Under nitrogen deprivation, filaments of the cyanobacterium *Anabaena* undergo a process of development, resulting in a one-dimensional pattern of nitrogen-fixing heterocysts separated by about ten photosynthetic vegetative cells. Many aspects of gene expression before nitrogen deprivation and during the developmental process remain to be elucidated. Furthermore, the coupling of gene expression fluctuations between cells along a multicellular filament is unknown. We studied the statistics of fluctuations of gene expression of HetR, a transcription factor essential for heterocyst differentiation, both under steady-state growth in nitrogen-rich conditions and at different times following nitrogen deprivation, using a chromosomally-encoded translational *hetR-gfp* fusion. Statistical analysis of fluorescence at the individual cell level in wild-type and mutant filaments demonstrates that expression fluctuations of *hetR* in nearby cells are coupled, with a characteristic spatial range of circa two to three cells, setting the scale for cellular interactions along a filament. Correlations between cells predominantly arise from intercellular molecular transfer and less from cell division. Fluctuations after nitrogen step-down can build up on those under nitrogen-replete conditions. We found that under nitrogen-rich conditions, basal, steady-state expression of the HetR inhibitor PatS, cell-cell communication influenced by the septal protein SepJ and positive HetR auto-regulation are essential determinants of fluctuations in *hetR* expression and its distribution along filaments. A comparison between the expression of *hetR-gfp* under nitrogen-rich and nitrogen-poor conditions highlights the differences between the two HetR inhibitors PatS and HetN, as well as the differences in specificity between the septal proteins SepJ and FraC/FraD. Activation, inhibition and cell-cell communication lie at the heart of developmental processes. Our results show that proteins involved in these basic ingredients combine together in the presence of inevitable stochasticity in gene expression, to control the coupled fluctuations of gene expression that give rise to a one-dimensional developmental pattern in this organism.

## Author Summary

Under prolonged nitrogen deprivation, one-dimensional filaments of the multicellular cyanobacterium *Anabaena* undergo a process of development, forming a pattern consisting of cells specialized for nitrogen fixation-heterocysts-, separated by a chain of about ten photosynthetic vegetative cells. The developmental program uses activation, inhibition, and transport to create spatial and temporal patterns of gene expression, in the presence of unavoidable stochastic fluctuations in gene expression among cells. Using a chromosomally-encoded fluorescent marker, we followed the expression of the important regulator HetR in individual cells along filaments, both under abundant nitrogen conditions as well as at different times after nitrogen deprivation. The results of our statistical analysis of these fluctuations illuminate the fundamental role that positive feedback, lateral inhibition and cell-cell communication play in the developmental program, not only after exposure to the external cue that triggers differentiation but also under non-inducing conditions. Furthermore our results establish the spatial extent to which gene expression is correlated along filaments.

## Introduction

In response to nitrogen deprivation, some nitrogen-fixing, photosynthetic cyanobacterial filaments such as those of the genera *Anabaena* and *Nostoc* undergo a process of development into a pattern consisting of single, specialized micro-oxic cells in which nitrogen fixation takes place-heterocysts-, separated by about 10–15 photosynthetic vegetative cells [1,2]. A one-dimensional, multicellular organism consisting of two types of cells with a clear division of labor is thereby formed. While neighboring cells can attain different developmental fates, cellular decisions may be driven by tiny differences in the concentrations of morphogens and other molecular species between cells [3]. These differences take place against the backdrop of the unavoidable cell-to-cell variability in gene expression between isogenic cells or noise [4,5]. Noise, extensively studied in unicellular organisms [6–8], has been shown to play a functional role in cellular decisions [9] and in the determination of developmental fates [10,11]. While noise may confer an advantage as an adaptive mechanism to changing environments [12], it must be tightly controlled during the development of a multicellular organism, in order to avoid its amplification and thus erroneous irreversible cell fates [13,14]. Unlike unicellular organisms, multicellular organisms can use cell-cell communication in order to control noise in gene expression. Evidence of such communication has been provided in the case of the *Drosophila* embryo, where spatial averaging in the level of the *hunchback* protein over ∼5 nuclei has been observed [15].

Intercellular communication is likely an important factor for determining heterocyst pattern formation [16–18]. Possible pathways for intercellular communication have recently been identified, and they include putative septal junction complexes of which the SepJ and FraC/FraD proteins are likely components [19,20].

The genetic cascade leading to heterocyst formation in cyanobacterial filaments is initiated by the NtcA transcription factor, which senses the low intracellular nitrogen concentration through the levels of 2-oxoglutarate [21]. The master regulator of differentiation HetR, which positively auto-regulates its own expression [22], is also induced early after nitrogen step-down in an NtcA-dependent way [23]. In contrast to those positive factors, an inhibitor of heterocyst differentiation at early times, PatS, and another inhibitor that is produced at later times, HetN, suppress the differentiation of vegetative cells into nitrogen-fixing heterocysts [18,24,25]. Both inhibitors share the amino acid motif ERGSGR in their sequence, which may act by inhibiting HetR [26–30]. Most work to date has focused on studying aspects of filament behavior following nitrogen deprivation, such as the changes in gene expression associated with the cellular differentiation decision, signal and metabolite transport along filaments, as well as the formation of the heterocyst pattern and its maintenance. In addition, statistical characterization of filaments following nitrogen deprivation has addressed primarily the fraction of heterocysts formed as well as the intervals between them, long after differentiation decisions have been made. The nature of the steady-state primordial fluctuations in gene expression along a filament under abundant nitrogen conditions, and those that build up on them at different times after combined nitrogen sources become scarce, eventually resulting in a fully developed pattern have not been studied to date.

In this paper we address these issues and characterize the noise of expression of the *hetR* gene, as well as the spatial correlations of expression between cells, in filaments of *Anabaena* sp. strain PCC 7120 (hereafter *Anabaena*) both under steady-state, abundant-nitrogen conditions, and at various times following nitrogen deprivation. To dissect the different contributions to variability, we studied expression fluctuations from a translational *hetR-gfp* fusion in wild-type filaments as well as in mutant backgrounds in which the PatS or HetN inhibitors are absent, backgrounds in which the transport between neighboring cells is potentially hindered, or when the positive auto-regulation of HetR is impaired. We found that key factors known to act during differentiation following nitrogen deprivation can also play a role before differentiation has been triggered, setting an underlying pattern of spatial correlations of gene expression along filaments, on which the developmental pattern may later be built. These correlations extend to two to three cells, setting the scale for cellular interactions along a filament. Moreover, we found evidence of the participation of an intercellular communication protein in setting these spatial correlations at specific times during differentiation.

## Results

### Experimental system

**Fig 1:**
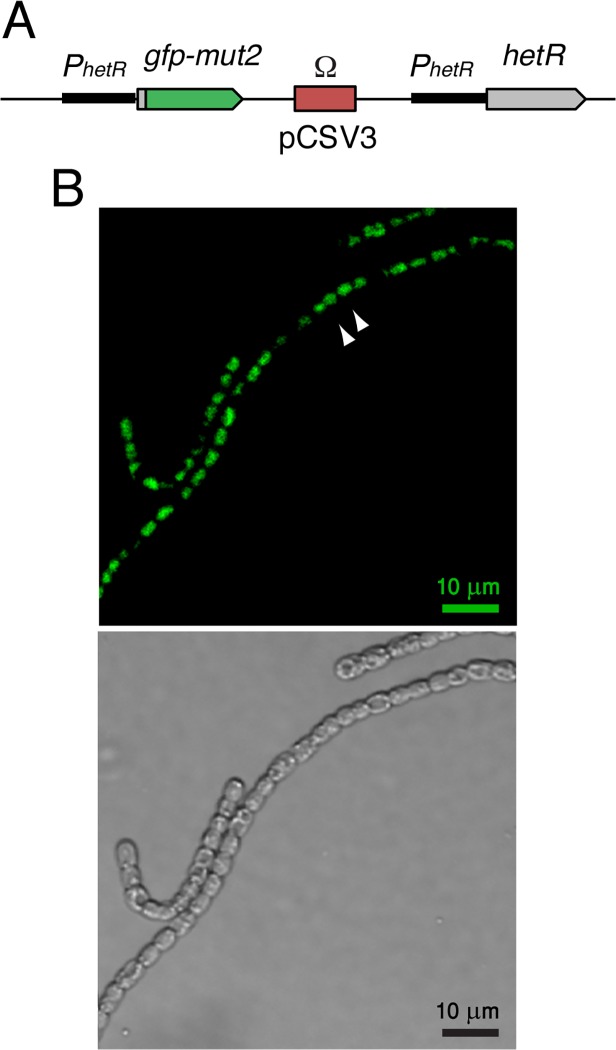
Expression of *hetR-gfp* in *Anabaena* filaments. **A** Scheme of the *hetR-gfp* fusion construct introduced in the genome of the WT and mutant *Anabaena* strains analyzed in this study. Plasmid pCSL68 bearing the *hetR-gfp* fusion in vector pCSV3 was incorporated into the genome of the recipient *Anabaena* strain by single recombination resulting in a duplication of the *hetR* promoter region and incorporation of pCSV3 (including the Ω fragment that encodes Sm^R^/Sp^R^). Hence, the *hetR-gfp* fusion is expressed from a complete *hetR* promoter, and a complete *hetR* gene can also be transcribed. The *hetR-gfp* fusion consists of the *hetR* promoter, the first 9 codons of *hetR*, a sequence encoding a tetra-glycine linker, and the *gfp-mut2* gene. All the strains were homozygous for the mutant chromosomes. For details see Materials and Methods. **B** (*top*) Typical snapshot of *hetR-gfp* fluorescence in a wild-type background, illustrating a cluster of cells (white arrows) exhibiting fluorescence levels above a given threshold. (*bottom*) Same field of view under bright light illumination.

Our study was conducted using a chromosomal *hetR-gfp* fusion, thereby avoiding any contribution to fluctuations stemming from cell-cell variability in plasmid copy number, which would preclude accurate evaluation of both noise and cell-cell correlations in expression [18]. To study expression of the *hetR* gene, we have constructed a *hetR-gfp* fusion in which a DNA sequence encoding a tetra-glycine linker and the *gfp-mut2* gene encoding a green fluorescent protein (GFP) were added after the 9^th^ codon of the *hetR* gene in a DNA fragment containing about 1 kb 5’ from the *hetR* gene and the 5’ terminal 27 bp of *hetR*. As a reporter, a fusion to a fragment of the N-terminal part of the protein was constructed rather than a traditional transcriptional fusion because with the N-terminal translational fusion, expression of the gene with all its transcription and translation start signals is tested. This construct, cloned in vector pCSV3 that bears an Sm^R^/Sp^R^ determinant (the Ω fragment [31]), was transferred by conjugation to different *Anabaena* strains with selection for resistance to streptomycin and spectinomycin. Recombination in the genomic region of homology results in the genomic structure shown in Fig. 1A, in which the fusion construct is placed downstream from the native *hetR* promoter and the *hetR* gene is located downstream from a 1-kb region that contains the whole *hetR* promoter [23,32]. Because the Ω fragment has transcriptional terminators at both its ends, no significant transcription comes out of pCSV3. The *hetR-gfp* fusion was transferred to wild-type *Anabaena* (WT) and mutant strains with deletions in the *patS* regulatory gene [33], in the *hetN* regulatory gene [27], in the *sepJ* gene encoding septal protein SepJ [19], and in the *fraC* and *fraD* genes encoding septal proteins FraC and FraD [20] (see Materials and Methods for details). In all the resulting strains, a native *hetR* gene is expressed. Additionally, the *hetR-gfp* fusion was transferred to a mutant lacking most of the *hetR* coding sequence, strain CSSC2 [34], permitting the study of *hetR-gfp* expression in the absence of a native HetR protein. As shown in Fig. 1B, readily appreciable green fluorescence was produced in the strain carrying the *hetR-gfp* fusion in a wild-type background. An important qualification for the interpretation of our results is that the GFP protein has been shown to remain in the cytoplasm of the producing cell [18,35]. GFPmut2 folds and matures within 5–30 min [36,37]. We will henceforth refer as WT or the corresponding gene mutations to the genetic backgrounds to which the *hetR-gfp* fusion has been added.

### Distributions *of hetR-gfp* expression under nitrogen-rich conditions

A typical image of WT filaments exhibiting expression from the translational *hetR-gfp* fusion in cultures with ammonium as a nitrogen source is shown in Fig. 1B. Basal expression from a transcriptional *hetR-gfp* fusion under similar conditions has already been reported [38].

**Fig 2:**
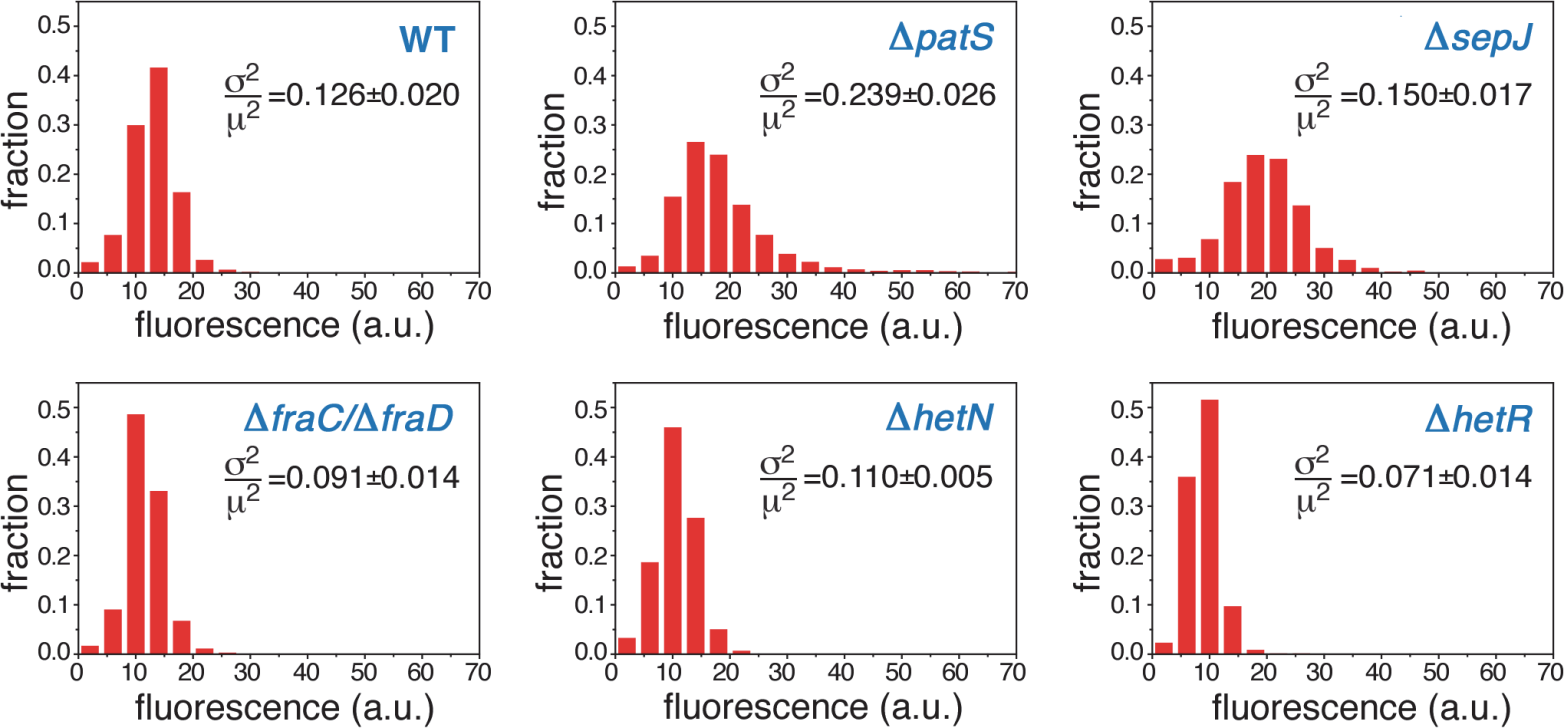
Expression of *hetR-gfp* along filaments under steady-state, nitrogen-rich conditions. Normalized histograms of cell fluorescence of *hetR-gfp* under nitrogen-rich conditions. The histograms, for the WT, Δ*patS*, Δ*sepJ*, Δ*fraC/*Δ*fraD*, Δ*hetN* and Δ*hetR* are comprised of typically ∼1500 cells. The value of the protein noise $\sigma_{p}^{2}/\mu_{p}^{2}$, where *σ*_*p*_ is the standard deviation and *μ*_*p*_ the mean of *hetR-gfp* fluorescence distributions over the filaments is shown in each histogram. The noise values represent an average over three independent runs, while errors denote standard errors.

Normalized histograms of cell fluorescence of *hetR-gfp* under steady-state nitrogen-rich conditions are shown in Fig. 2 for a typical experimental run. The histograms of the Δ*patS* and Δ*sepJ* mutants are significantly broader than the WT histogram, and their respective means ±std (18±8 and 19±7) larger than the WT’s (13±4). The histogram of Δ*patS* exhibits a particularly long tail of high fluorescence values, which is consistent with a possible lack of inhibition of *hetR* expression. In contrast to the Δ*patS* and Δ*sepJ* strains, mutants lacking either HetN, or the cell-cell junction proteins FraC/FraD, yield fluorescence distributions that are similar to that of the WT background. These similarities and differences are succinctly captured quantitatively by the Earth Mover’s Distance or EMD [39] (Table 1), a cross-bin metric to compare pairs of histograms (Eq. 2, Materials and Methods). The smaller the EMD, the larger is the similarity between the histograms. The histogram corresponding to the Δ*hetR* strain lacking HetR positive auto-regulation is narrower than that of the WT and all the other mutants, and the filaments display fluorescence values that are typically smaller than all other strains. This suggests that autoregulation of *hetR* is also at play under nitrogen-replete conditions.

**Table 1:** The Earth Mover’s distance EMD between pairs of histograms of HetR fluorescence from the *hetR-gfp* construct under nitrogen-replete conditions.

EMD	WT	Δ*hetN*	Δ*fraC/*Δ*fraD*	Δ*sepJ*	Δ*patS*
**WT**	0	0.58±0.04	0.35±0.03	1.61±0.06	1.26±0.06
Δ***hetN***		0	0.24±0.03	2.18±0.06	1.84±0.06
Δ***fraC/***Δ***fraD***			0	1.96±0.06	1.60±0.06
Δ***sepJ***				0	0.55±0.05

The distances, computed using Eq. 2, correspond to the histograms for the WT and Δ*patS*, Δ*hetN*, Δ*sepJ* and Δ*fraC/*Δ*fraD* strains shown in Fig. 1. Higher similarity between histograms corresponds to a lower EMD value. Errors have been estimated from 1000 bootstrap samples.

### Noise under nitrogen-rich conditions

The steady-state cell-to-cell variability in *hetR* expression, also called noise, is quantified by the non-dimensional ratio $\sigma_{p}^{2}/\mu_{p}^{2}$, where *σ*_*p*_ is the standard deviation and *μ*_*p*_ the mean *hetR-gfp* fluorescence distributions over filaments. The noise characterizing the different strains under the steady-state conditions of our experiments is shown in each panel in Fig. 2. The noise of *hetR-gfp* in the Δ*patS* background is by far the largest among all strains, consistent with the wide range of *hetR-gfp* levels this strain displays (Fig. 2). The level of noise characterizing Δ*sepJ* filaments lies intermediate between those of the WT and the Δ*patS* strains. In contrast, the strain lacking HetR exhibits the smallest level of noise. Lastly, the noise levels of expression of *hetR-gfp* in the strains Δ*hetN* and Δ*fraC/*Δ*fraD* are somewhat smaller from those of the WT background. We conclude that cell-to-cell fluctuations of the master regulator HetR in *Anabaena* are complex and include essential contributions from the developmental network even when the latter is not induced.

### Correlations in the expression of HetR-GFP between neighboring cells under nitrogen-replete conditions

**Fig 3:**
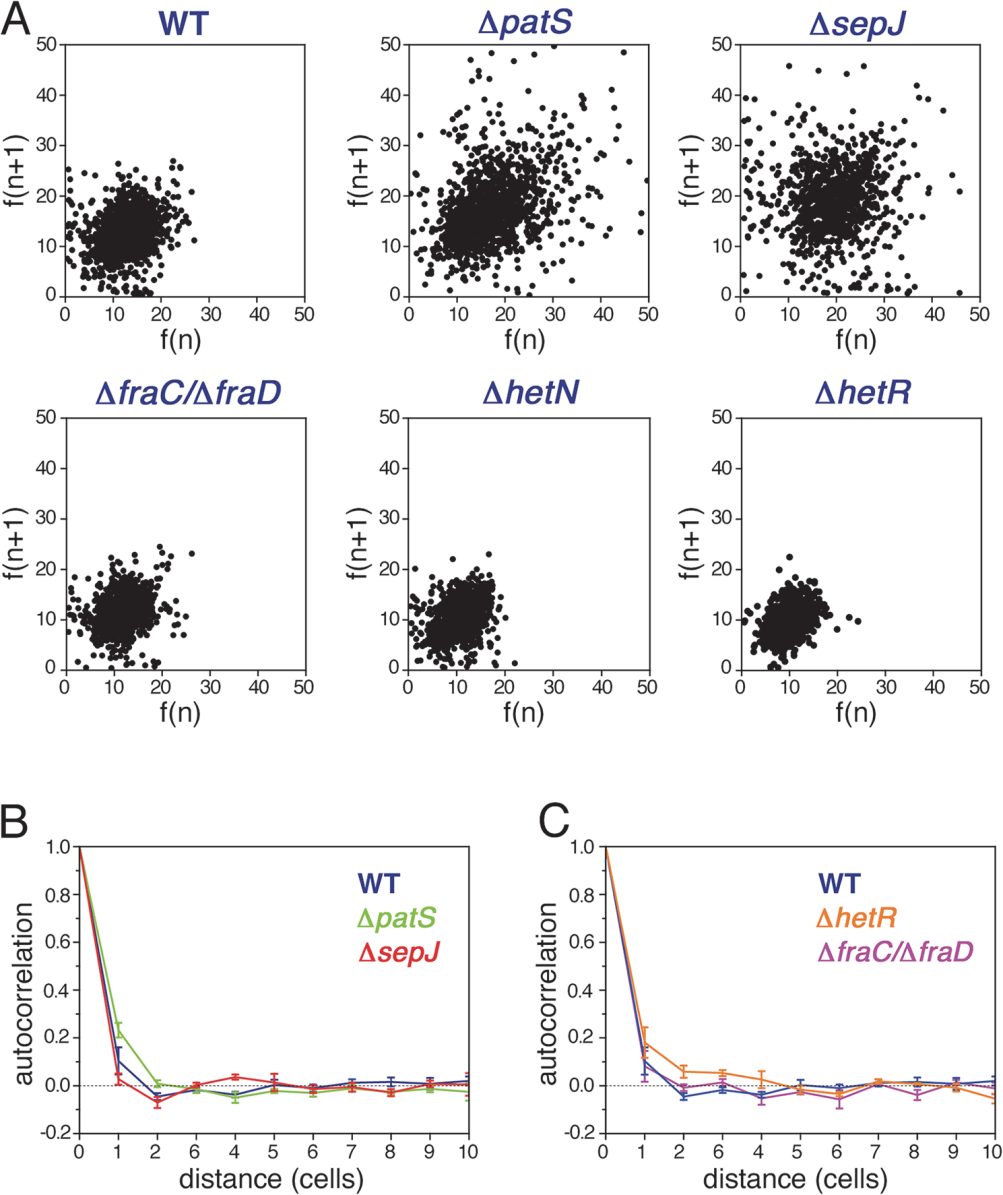
Correlated fluctuations of *hetR-gfp* expression along filaments. **A** Scatter plots of the fluorescence of the n+1-th cell in a filament *f(n+1)* as function of the fluorescence *f(n)* of its nearest neighbor cell, for the WT, Δ*patS*, Δ*sepJ*, Δ*fraC/*Δ*fraD*, Δ*hetN*, and Δ*hetR* mutant strains. **B** Autocorrelation of HetR-GFP fluorescence along filaments for the WT, Δ*patS*, and Δ*sepJ* backgrounds. The traces represent means of three (WT, Δ*patS*) and two (Δ*sepJ*) experiments, while the error bars represent the respective standard errors. The negative bias due to the estimation of the mean filament fluorescence in the calculation of the covariance of the autocorrelation has been corrected [40] (Materials and Methods). **C** Autocorrelation of HetR-GFP fluorescence along filaments for the WT, Δ*hetR* and Δ*fraC/*Δ*fraD* backgrounds. The negative bias was corrected as in B. The traces represent means of three independent experiments, while the error bars represent the respective standard errors.

In contrast to unicellular organisms in which fluctuations in gene expression between different cells are independent [4], we expected to find correlations between the fluctuations in *hetR-gfp* expression in *Anabaena* along filaments (Fig. 1B). Spatial correlations are clearly brought out by scatter plots of the fluorescence *f(n+1)* of the *n+1*-th cell in a filament as function of the fluorescence *f(n)* of its nearest neighbor along all filaments, as shown for the different strains in Fig. 3A. The most salient feature in the scatter plots is the large spread of points in the cases of Δ*patS* and Δ*sepJ*, relative to the WT, Δ*hetN*, Δ*hetR* and Δ*fraC/*Δ*fraD*, for which the scatter plots are rather similar. This similarity indicates that the effect of the basal activity of HetN on *hetR-gfp* expression is not significant under combined nitrogen, and that the possible distribution of a regulatory signal along filaments is not severely affected in the Δ*fraC/*Δ*fraD* mutant. The cigar shape of the scatter for Δ*patS* (Pearson coefficient = 0.40) reveals a larger statistical tendency for nearest neighbors cells to have similar fluorescence than in the WT (Pearson coefficient = 0.25). In contrast, the observed tendency of some highly fluorescent cells to have weakly fluorescent neighbors in Δ*sepJ* filaments (Pearson coefficient = 0.05), suggests that expression fluctuations in this strain are less constrained than in the WT. However, the non-trivial distribution of points in the scatter plot for Δ*sepJ* hints that cells are not fully independent.

To measure the spatial extent over which fluctuations in different cells are correlated, we calculated the autocorrelation function of the HetR-GFP fluorescence *g*(*n*) as a function of the distance *n* between cells for each filament, and then averaged over all the filaments of the specific strain in the experimental run:

$$g\left(n\right)={\langle \frac{1}{\left(L_{f}-n\right)}\sum_{m}\frac{\left(F(n+m)-\mu_{f})(F(m)-\mu_{f}\right)}{\sigma_{f}^{2}}\rangle }_{\mathrm{filaments}}$$ (1)

Here *F*(*n*) denotes the fluorescence of the *n*-th cell in a filament, *μ*_*f*_ is the mean fluorescence per cell in a given filament, $\sigma_{f}^{2}$ the respective variance, *L*_*f*_ the filament length and the index m runs over all cells in a given filament. The autocorrelation, compensated for a negative statistical bias [40] (see S1 Fig.), averaged further over three independent experimental runs is plotted for the WT, Δ*patS* and Δ*sepJ* strains in Fig. 3B, and for the WT, Δ*fraC/*Δ*fraD* and Δ*hetR* in Fig. 3C. Within each run, the correlation functions for the different strains display the same behavior relative to one another, as reflected by the small standard errors. Therefore, the differences between the correlation functions corresponding to the different strains for *n* ≤ 3 observed in Fig. 3B, though small, are significant. The marked differences between the scatter plots of the different strains (Fig. 3A), whose corresponding Pearson correlation coefficients are nothing but the values of *g*(*n*) for *n* = 1, underscore this significance. While the decay of *g*(*n*) with distance *n* for the Δ*patS* strain is slower than for the WT, it is faster for the Δ*sepJ* strain. In contrast to the Δ*sepJ* strain, the correlation function corresponding to Δ*fraC/*Δ*fraD* strain is very similar to that of the WT. We note that filament fragmentation cannot be invoked to account for any of these results since all the filament segments in our analysis are 7 cells or longer, larger than the spatial extent over which fluctuations in expression are correlated. Irrespective of the strain, the behavior illustrated in Fig. 3B and 3C indicates that gene expression between cells under nitrogen-rich conditions is correlated up to distances of two to three cells.

The small fluctuations of *hetR-gfp* expression that Δ*hetR* strain filaments display (Fig. 2) led us to expect a slower decay of its autocorrelation relative to the WT, as is indeed observed in Fig. 3C. Further analysis reveals that in this strain, differences of *hetR-gfp* expression between cells within individual filaments are so small that the principal contribution to expression variation across the cell population comes from inter-filament differences in expression. To quantify this, we first calculated the means of the fluorescence of all cells within each filament, and then computed the mean *μ*_*f*_ and standard deviation *σ*_*f*_ of these fluorescence means over all filaments to obtain the noise $\sigma_{f}^{2}/\mu_{f}^{2}$. We then compared $\sigma_{f}^{2}/\mu_{f}^{2}$ to ${\langle \sigma_{p}^{2}/\mu_{p}^{2}\rangle }_{f}$, obtained by calculating first the noise for each filament and then averaging over filaments. The ratio between $\sigma_{f}^{2}/\mu_{f}^{2}$ and ${\langle \sigma_{p}^{2}/\mu_{p}^{2}\rangle }_{f}$ is 23±1% (mean±se) for all strains except for Δ*hetR*. In contrast, this ratio is equal to 61% for the Δ*hetR* strain. Thus, within Δ*hetR* filaments, cells display high similarity in *hetR-gfp* expression.

### Spatial range of correlations in *hetR-gfp* expression

**Fig 4:**
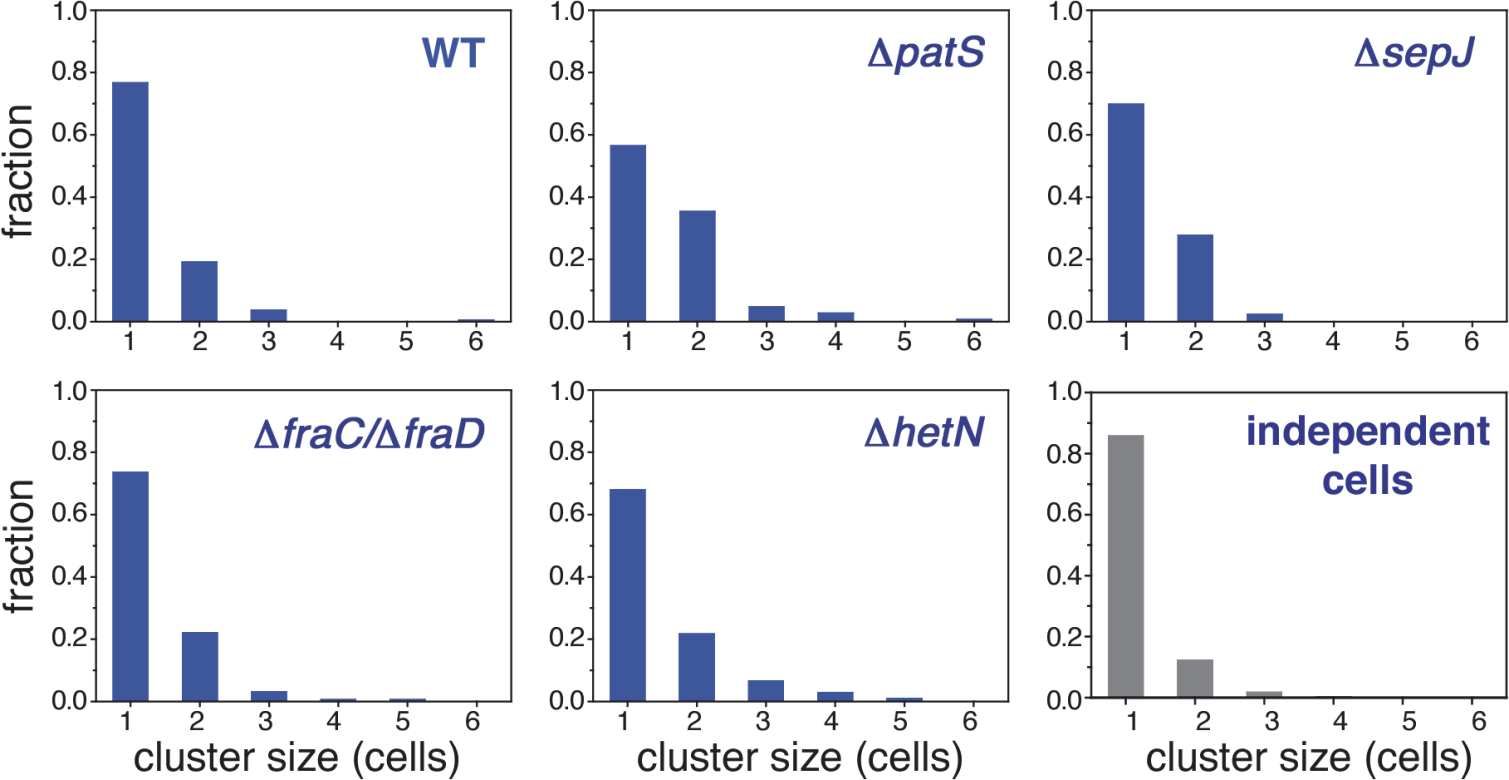
Higher order correlations of *hetR-gfp* expression along filaments. Histograms of sizes of clusters of contiguous cells along a filament whose fluorescence is above a threshold value for the WT, Δ*patS*, Δ*sepJ*, Δ*fraC/*Δ*fraD* and Δ*hetN*. The threshold chosen here corresponds to the upper 15 percentile of cells in the fluorescence histograms in Fig. 2. The histogram in gray is the result of a computer simulation of 1000 finite strings of 20 cells in which ones were thrown *independently* with probability p = 0.15 among zeroes.

A two-point correlation function such as *g*(*n*) does not capture higher order correlations such as the recurrent observation of clusters of contiguous cells exhibiting GFP fluorescence values from *hetR-gfp* above a given threshold, as illustrated in the typical snapshot of WT in Fig. 1B. To investigate higher-order spatial correlations between contiguous cells, we made a binary representation of each filament by choosing a threshold value of fluorescence and dividing cells into two classes: those whose fluorescence is either smaller or larger than the threshold. Cells with fluorescence levels larger than the threshold were assigned the value of 1, while cells with smaller fluorescence levels than the threshold were assigned the value 0. We then evaluated the length of each cluster of contiguous 1’s and the distribution of cluster sizes. We show in Fig. 4 histograms of cluster sizes obtained when setting the fluorescence threshold as the value defining the upper 15 percentile of cells, for each of the histograms of Fig. 2 (this choice yields fluorescence threshold values that are close to those obtained when choosing the threshold as the value corresponding to the mean-plus-standard deviation of each distribution). The cluster size histograms for the WT, Δ*hetN* and Δ*fraC/*Δ*fraD* strains decay the fastest, whereas Δ*patS* the slowest. To make a quantitative comparison between these histograms we calculated the statistic *X* ^*2*^ (Eq. 3, Materials and Methods), which has approximately a *χ* ^*2*^ distribution [41]. A perusal at Table 2 indicates that there is a significant likelihood that the cluster size histograms of the WT, Δ*hetN* and Δ*fraC/*Δ*fraD* come from the same distribution. In contrast, the cluster size histograms of the WT and Δ*patS* differ significantly. The cluster size histogram of Δ*sepJ* is as different from that of the WT as from the Δ*patS* histogram. Together, these facts highlight again the fundamental role that the PatS inhibitor plays in determining the fluctuations along a filament.

**Table 2:** Statistical comparison between histograms of cluster sizes for the different strains.

strain vs strain	*X* ^*2*^	p-value
WT vs Δ*hetN*	2.23	0.33
WT vs Δ*fraC/* Δ*fraD*	0.56	0.76
WT vs Δ*patS*	13.65	0.001
WT vs Δ*sepJ*	3.36	0.19
Δ*patS* vs Δ*sepJ*	4.22	0.12

Pairwise statistical comparison between strains using the *X* ^2^ statistic (Eq. 3, Materials and methods). The data correspond to the histograms shown in Fig. 4.

### Comparing the fluctuations of cells in a WT filament with fluctuations in filaments consisting of independent cells

An alternative way to bring out the correlated character of *hetR-gfp* expression fluctuations along a filament is to measure the extent to which real filaments differ from filaments consisting of independent cells. To this end we simulated filaments of independent cells by generating finite strings of 20 cells, with ones thrown *independently* with probability p = 0.15 among zeroes (the Bernoulli problem, which in the infinite string limit yields the negative binomial distribution). We then calculated from the simulated filaments a cluster size histogram (Fig. 4). The EMD distance (Eq. 2, Materials and Methods) between this histogram and the histograms corresponding to the measured strains is shown in Table 3. We find that under nitrogen-replete conditions, the EMD distance between the independent-cell cluster size histogram and those of the WT and the strains in which septal proteins have been deleted are the smallest. In contrast, the EMD distance between the independent-cell cluster size histogram and that corresponding to Δ*patS* is the largest.

**Table 3:** EMD distance between cluster size histograms of the different strains and histograms obtained from simulations of independent cells.

strain	EMD with simulations
WT	0.12±0.02
Δ*patS*	0.40±0.03
Δ*hetN*	0.31±0.06
Δ*sepJ*	0.17±0.03
Δ*fraC/*Δ*fraD*	0.16±0.02

EMD distance (Eq. 2, Materials and Methods) between the cluster size histogram of independent cells obtained from computer simulations and the cluster size histograms corresponding to the WT, Δ*patS*, Δ*hetN*, Δ*sepJ* and Δ*fraC/* Δ*fraD* strains shown in Fig. 4. Errors have been estimated from 10000 bootstrap samples.

### Non-stationary fluctuations following nitrogen deprivation

**Fig 5:**
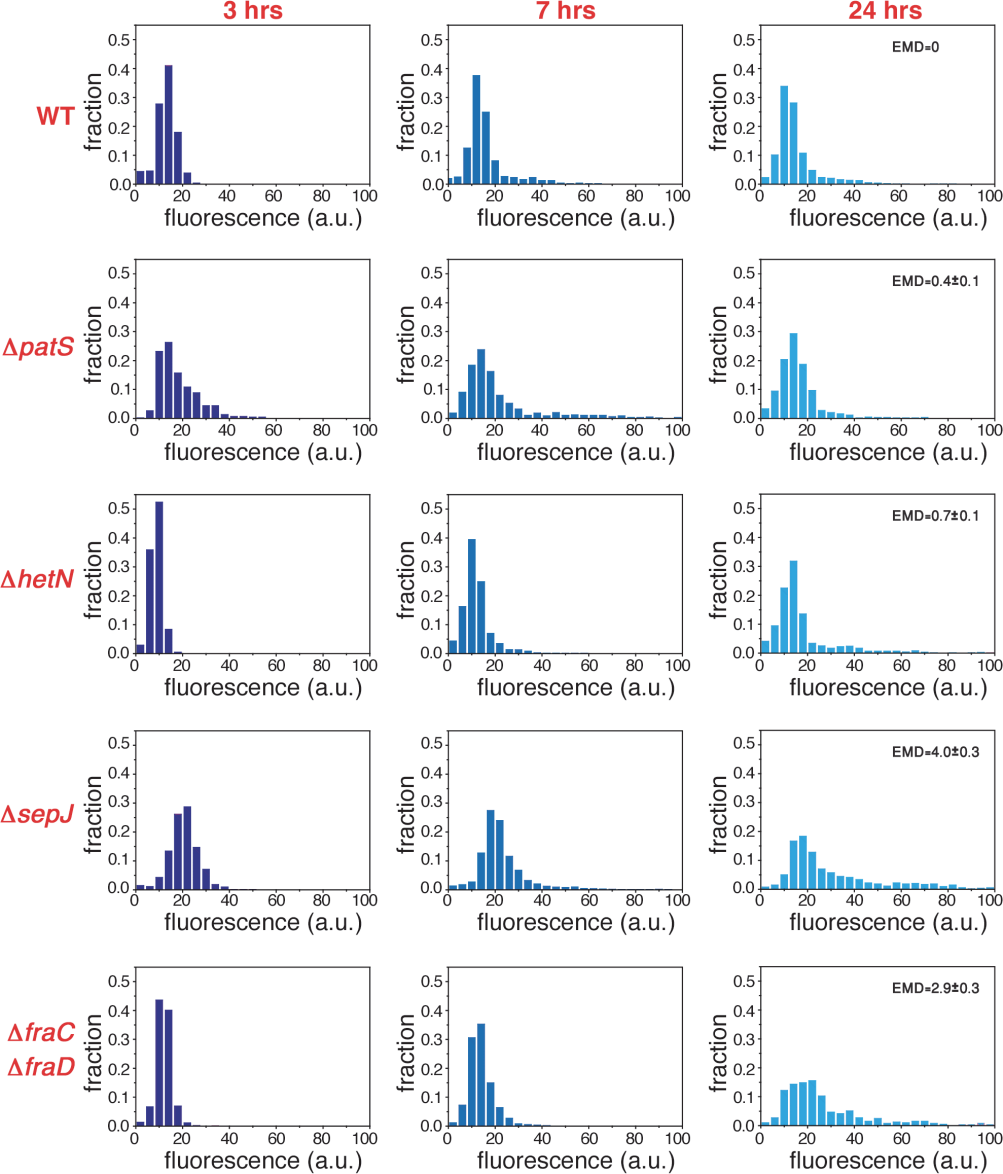
Non-stationary distributions of expression fluctuations of *hetR-gfp* following nitrogen deprivation build up from fluctuations under nitrogen-replete conditions. Normalized histograms of cell fluorescence of *hetR-gfp* 3, 7 and 24 hours after nitrogen step-down (different color hues), for the WT, Δ*patS*, Δ*hetN*, Δ*sepJ* and Δ*fraC/*Δ*fraD* strains. The EMD distance between the WT and the indicated mutants is shown in each panel in the case of measurements taken 24 hours after nitrogen deprivation. The errors in EMD were derived from 10000 bootstrap samples.

**Fig 6:**
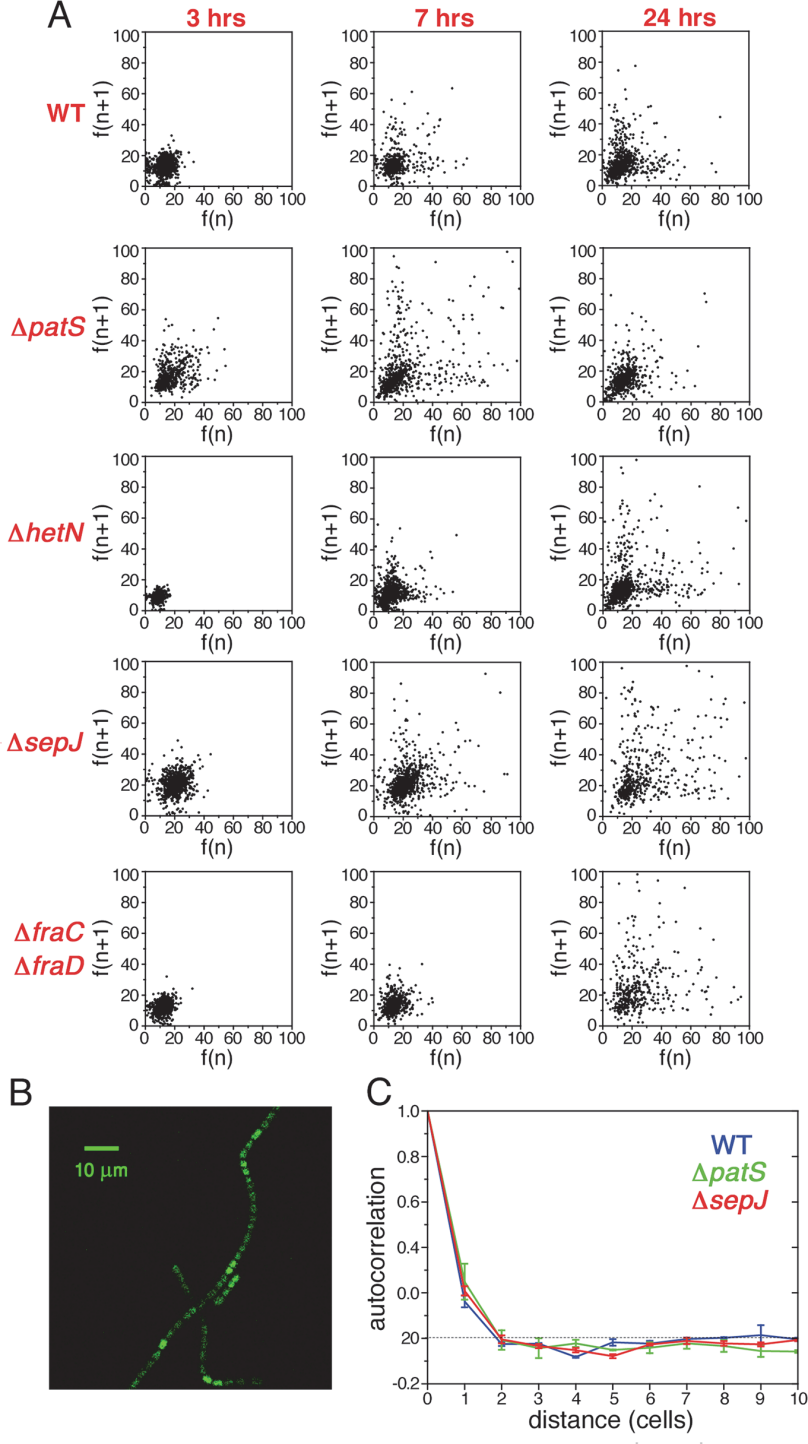
Non-stationary correlations between the expression fluctuations of *hetR-gfp* in adjacent cells following nitrogen deprivation. **A** Scatter plots of the fluorescence of the n+1-th cell in a filament *f(n+1)* as function of the fluorescence *f(n)* of its nearest neighbor cell for the WT and the indicated mutant strains, 3, 7 and 24 hours after nitrogen deprivation. **B** Typical snapshot of *hetR-gfp* fluorescence in a wild-type background 7 hours after nitrogen step-down, illustrating clustered expression from *hetR-gfp*. **C** Autocorrelation of HetR-GFP fluorescence along filaments for the WT, Δ*patS*, and Δ*sepJ* backgrounds, 7 hours after nitrogen step-down. The traces represent means of two experiments, while the error bars represent the respective standard errors.

To explore the possibility that the fluctuations in gene expression measured under nitrogen-replete conditions provide the background over which cell-to-cell variations build up during the development process, we have carried out an analysis of fluctuations of *hetR-gfp* expression at different times following nitrogen deprivation. Histograms of fluorescence values and scatter plots of nearest neighbors three hours following nitrogen deprivation (Figs. 5 and 6), display similar behavior to the corresponding histograms and scatter plots measured under nitrogen-replete conditions. However, seven hours after nitrogen step-down, changes in the spatial distribution of fluorescence values are clearly discernible: there is a marked tendency for some pairs of adjacent cells to display high and low fluorescence values, in contrast to the similar fluorescence values displayed by nearest-neighboring cells three hours after nitrogen step-down (Fig. 6A). It is also noteworthy that 7 hours after nitrogen step-down the behaviors of Δ*sepJ* and Δ*fraC/*Δ*fraD* are quite different: whereas Δ*sepJ* filaments have cells in which *hetR-gfp* is highly expressed and there is an anti-correlation between the fluorescence values of *hetR-gfp* in adjacent cells, the fluorescence values in Δ*fraC/*Δ*fraD* filaments are lower and comparable to the WT. Furthermore, in the Δ*fraC/*Δ*fraD* filaments, no anticorrelation is observed between the expression in nearest-neighbor cells as at 3 hours. The behaviors displayed by Δ*sepJ* and Δ*fraC/*Δ*fraD* strains become similar 24 hours after nitrogen step-down: both strains display long tails of high fluorescence values in their histograms and nearest-neighbor pairs of cells behave in a comparable fashion, in contrast to the different behavior displayed by the two strains in assays using non-physiological fluorescent molecules [20]. Moreover, the behavior of these mutants, which lack putative septal proteins, differs significantly from both the WT and the mutants Δ*patS* and Δ*hetN*. Indeed, the EMD values between the WT and all the mutants, displayed in the panels corresponding to 24 hours (Fig. 5) confirm this. Finally, we note that in the Δ*hetR* strain, the expression of *hetR-gfp* following nitrogen step-down does not change significantly from that under nitrogen-replete conditions (S2 Fig.).

To capture the changes in expression fluctuations when a pattern commences to emerge, as illustrated in Fig. 6B, we have calculated the autocorrelation as a function of distance *g*(*n*) for the WT, Δ*patS* and Δ*sepJ* strains, at 7 hours after nitrogen step-down (Fig. 6C). This time is close to the time of commitment to differentiation [24]. Remarkably, there is a significant increase in correlation both for the WT and the Δ*sepJ* strains for *n* ≤ 2, while not for the Δ*patS* strain, when compared to ammonium-replete conditions. The respective autocorrelation functions are undistinguishable within experimental error, in spite of noticeable differences in the respective scatter plots.

## Discussion

In an organism with no specific limit to the number of its cells and on which morphogen gradient fields are not imposed from the outside such as in *Drosophila*, a developmental pattern can arise from fluctuations in gene expression and activation-inhibition mechanisms such as originally envisioned by Turing [42]. Variations in gene expression between cells play a key role in determining cellular decisions during developmental processes [14,43]. Here we have characterized the variability and spatial distribution of *hetR-gfp* expression levels along *Anabaena* filaments, both under nitrogen-replete and nitrogen-poor conditions. We have further calculated for the first time correlations between the expression levels in cells along filaments and the spatial extent to which these levels are coupled. Several important features emerge from our study.

**Fig 7:**
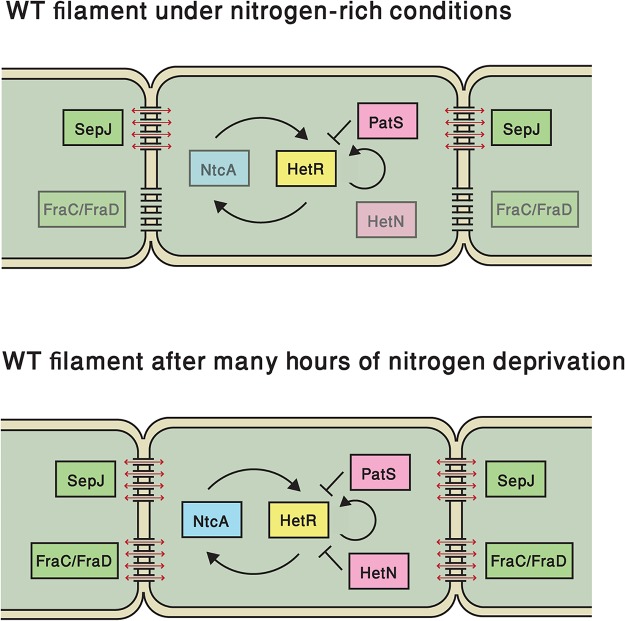
Elements that regulate *hetR* expression in this study, both under nitrogen-replete conditions and following nitrogen deprivation. Key regulatory elements of the master regulator HetR (yellow) in the developmental network, both under nitrogen-replete conditions (top) or following nitrogen deprivation (bottom): pink elements represent inhibitors of HetR function, while green elements denote septal proteins involved in cell-cell communication SepJ and Fra C/D. NtcA (blue) detects nitrogen status in the cell. Faint boxes represent elements that are present but may not have effects on *hetR* expression fluctuations at the indicated time. Black lines ending either in arrows or bars denote positive or negative interactions respectively, which may not necessarily be direct. Red arrows denote intercellular transport of diffusing species, which may include PatS or a derived peptide or HetN.

Notably, we found that the variability in the basal state of *hetR* expression under nitrogen-replete conditions is governed to a large extent by important factors of the developmental network, even if the latter is not induced. Many of our experimental observations support the notion of basal expression of a PatS-derived peptide and/or its transport between cells. First, the average expression level and the variability in the Δ*patS* strain are higher than in the WT. While the higher mean in the Δ*patS* strain points to an inhibitory effect of PatS, the broader distribution of expression can be accounted for by the fact that the negative feedback loop of PatS on HetR is broken. Second, the spatial autocorrelation of expression levels as function of the separation between cells decays slower in the Δ*patS* strain than in the WT, showing that the effect of basal PatS expression is to anti-correlate the expression of *hetR-gfp* between neighboring cells. Third, the clusters of contiguous cells exhibiting *hetR-gfp* expression above a threshold are larger in the Δ*patS* strain than in the WT. Together, these analyses support the notion that PatS or a derivative thereof is transferred between neighboring cells, consistently with results obtained with filaments that have already sensed nitrogen deprivation [18,30,33]. In contrast, basal expression of the long-term inhibitor HetN, if significant, does not show detectable effects on the expression of the *hetR-gfp* translational fusion and its fluctuations under combined nitrogen-rich conditions. At late times following nitrogen step-down, the effects of deleting *patS* or *hetN* are similar as seen in the respective fluorescence histograms. This is in line with the late-time activation of this gene for pattern maintenance purposes [25,27] (see Fig. 7).

We investigated two mutant strains, Δ*sepJ* and Δ*fraC/*Δ*fraD*, both of which lack proteins that are thought to contribute to intercellular septal junction complexes and cell-cell communication [19,20,44]. Specifically, we inquired whether the coupling of expression fluctuations of *hetR-gfp* between neighboring cells is impaired in these mutants, and if so, to what extent expression fluctuations in neighboring cells are independent of one another. We found that the Δ*sepJ* mutant’s behavior is markedly different from the wild type both under nitrogen-replete conditions, as well as at early and late times after nitrogen deprivation. The putative cell-cell transport of an active PatS-derived peptide would account for the behaviors of the Δ*sepJ* and the Δ*patS* strains under nitrogen-rich conditions. In the Δ*sepJ* strain, transport hindrance of such peptide does not smooth out *hetR-gfp* expression fluctuations between cells, thereby increasing cell-to-cell variability relative to the WT, as observed. The transport of a PatS-derived signal may also account for the slower decay of the autocorrelation function in the Δ*patS* background relative to the WT, while in the Δ*sepJ* strain it decays faster under nitrogen-rich conditions. We stress that the nature of the regulators transferred through the SepJ-containing septal junction complexes is as yet unknown and remains to be directly established. However, the behaviors of the Δ*patS* and Δ*sepJ* strains observed in the present work are consistent with cell-cell transfer of the active PatS peptide through SepJ-containing septal junction complexes. It has been recently reported that inactivation of *sepJ* caused a significant decrease in the signal range of *hetN* expressed from source cells via an inducible promoter on plasmids, but not of *patS* [45]. The differences between these results and ours may be reconciled by the prolonged production of PatS in that study, whereas our results reflect native conditions.

In contrast to Δ*sepJ*, the Δ*fraC/*Δ*fraD* mutant strain’s behavior is very similar to the WT, both under nitrogen-replete conditions and at early times after nitrogen step-down (e.g at 7 hours). This is surprising given that FraC and FraD assist in focusing SepJ to cell-cell junctions [44]. It is possible that in a Δ*fraC/*Δ*fraD* background, the SepJ molecules still present in the septum suffice to allow the transfer necessary to establish the observed correlations under combined nitrogen conditions [20]. In contrast, the behavior of the Δ*sepJ* and Δ*fraC/*Δ*fraD* mutant strains becomes comparable 24 hours after nitrogen step-down. These data suggest that FraC/FraD proteins are involved in the late time transport of specific metabolic and/or regulatory factors that may be different from those transported by SepJ, negatively impacting *hetR* expression in neighboring cells (Fig. 7). Previous transport assays on both Δ*sepJ* and Δ*fraC/*Δ*fraD* mutant strains using calcein or 5-carboxyfluorescein have shown differences in the intercellular transfer of these fluorescent tracers [20]. Although deletion of septal proteins could be expected to result in filaments in which gene expression fluctuations in cells behave independently, we found that correlations consistently persist. This is supported by the fact that none of the autocorrelation functions corresponding to the different genetic backgrounds tested display behavior expected for filaments of independent cells.

The spatial autocorrelation function of *hetR-gfp* expression along filaments shows that correlations between neighboring cells die out after two to three cells, a range of correlations that is shorter than the characteristic scale of developmental patterns. This sets the scale for cellular interactions along a filament under nitrogen-replete conditions. We stress that the dominant part of the observed correlations must derive from intercellular molecular transfer: had the measured correlations been due exclusively to cell division, there would be no difference between the autocorrelation functions pertaining to the different mutant strains. No differences in growth under permissive conditions (in the presence of ammonium) between the mutants have been noticed. Moreover, following nitrogen deprivation, our experiments do not reach steady diazotrophic growth under which differences in growth rate among mutants would be observed. Importantly, at intermediate stages after the developmental cue e.g. 7 hours, this correlation length has not appreciably changed. For comparison, the diffusion gradients of a PatS-derived peptide, a candidate factor mediating correlations between cells were recently measured 8 hours after nitrogen step-down, yielding about five cells [33]. Note that *hetR* gene expression levels are not a proxy for a PatS signal. Interestingly, evidence for the transfer of a HetN-derived signal with a range of approximately 10 cells, measured in filaments engineered to produce HetN from an inducible promoter, has recently been reported [45]. Induction for 72 hours resulted in HetN production from a number of contiguous cells, most likely with higher levels than in a native system, which may explain the larger signal range reported. The direct evidence for correlated expression between neighboring cells in *Anabaena*, revealed here for the first time, precludes the usual decomposition of noise into intrinsic and extrinsic components that is common in studies of unicellular organisms such as bacteria and yeast [4,46]. Another manifestation of the correlations between cells is the expression of *hetR-gfp* in contiguous cells along filaments. Our observations show that these clusters already exist under nitrogen-rich conditions, whereas previous investigations had shown that the differentiation process initiates in small clusters of cells that are later resolved into single heterocysts, after the environmental cue is given [24].

A mutant lacking *hetR* exhibits a much lower level of fluctuations of *hetR-gfp* expression, both under nitrogen-rich and nitrogen-poor conditions. These results can be accounted for by an attribute of HetR, namely auto-regulation, a mechanism that enhances fluctuations [47,48], which is known to operate under nitrogen deprivation [22]. This notion is supported by the significant reduction in noise in the Δ*hetR* strain, the slow decay of the corresponding autocorrelation function, and by the fact that the distribution of fluorescence values does not change appreciably following nitrogen step-down. Together, these findings suggest that positive auto-regulation is essential to build up the large levels of HetR needed for progression of differentiation in some cells and not in others, consistent with the lack of heterocyst formation in this mutant [22,49].

Our measurements of the statistical properties of fluctuations in gene expression of *hetR-gfp* well after the developmental network has sensed nitrogen step-down, but possibly before a final commitment has been established show that the primordial fluctuations under nitrogen-rich conditions and those at early times after nitrogen step-down, e.g. 3 hours, are statistically similar. This suggests that there is continuity between the two, and fluctuations found once the developmental network has been triggered, build up from those under nitrogen-rich conditions. At later times, 7 hours after nitrogen step-down, alterations in the spatial pattern of expression fluctuations can be discerned. The significant increase observed at small distances in the autocorrelation functions for the WT and Δ*sepJ* strains points to enhanced, concerted *hetR* expression in small subsets of contiguous cells as a dominant feature. This effect may result in part by the amplification of *hetR* clusters of high expression already existing under nitrogen-replete conditions, through positive auto-regulation. In contrast, the correspondingly small increase of the autocorrelation function for Δ*patS* may be due to the fact that clusters of high expression in this strain were already broader under nitrogen-replete conditions, given the lack of inhibition by PatS. A two-stage model of development in heterocyst-forming cyanobacteria has been proposed [50]. This model posits that during a first stage, only a subset of cells are transiently more likely than others to initiate differentiation prior or at the signal for nitrogen starvation [51]. During the second stage-competitive resolution- the competition between *hetR* expression and a PatS-derived peptide selects one cell among a cluster of contiguous cells of enhanced *hetR* expression to differentiate. The observed increase in positive correlations at small distances in the correlation functions provides direct statistical support for the existence of the second stage, namely competitive resolution.

To sum up, we have provided direct evidence for the coupling of gene expression fluctuations between cells in *Anabaena* filaments, prior and following nitrogen step-down. The range over which expression in neighboring cells is correlated, two to three cells, is smaller than the characteristic scale of fully developed patterns. Our results show that fluctuations can build up from an underlying pattern of expression under nitrogen-replete conditions in which important regulators are already expressed, and evolve to intermediate states in which fluctuations are positively correlated in clusters of cells. These latter resolve after commitment into single heterocysts using lateral inhibition, supporting a two-stage model of development. Therefore, expression fluctuations are key determinants in the decision of cells whether to differentiate or not, together with other factors such as cell division and biased inheritance of specific factors [51], as well as the marked tendency for heterocysts to form at the ends of finite filaments, thereby imposing a strong constraint on the phase of the patterns that form. We have further shown by a novel approach that important information can be garnered about functional properties of specific components of a developmental network, by characterizing the expression fluctuations of a master regulator under steady-state, non-induced conditions and after induction. The analysis brings out the different roles and specificity of inter-cellular communication proteins in setting spatial correlations, before and during development. Future studies of the coupling of gene expression fluctuations between cells in *Anabaena* and other organisms, using similar approaches, will shed light on other functions and on the way signals propagate during developmental processes as they occur.

## Materials and Methods

### Strains

*Anabaena* sp. (also known as *Nostoc* sp.) strain PCC 7120 and derived strains were grown photoautotrophically as described previously[33]. For construction of a fusion of *hetR* to the *gfp-mut2* gene, a DNA fragment was amplified using *Anabaena* DNA as template and primer pair alr2339–12/alr2339–36, the latter containing a sequence encoding a GGGG linker preceded by a NheI site. (Primer sequences: alr2339–12, 5’ AACTCTGGACTTCTGGCT 3’; alr2339–36, 5’ GCTAGCACCTCCACCGCCCTTGATCAGATCGATGT 3’.) The PCR product was cloned in pSPARK (Canvax), corroborated by sequencing and transferred to pCSAL33, which bears the *gfp-mut2* sequence preceded by a NheI site, and the resulting *hetR*-*gfp-mut2* construct was transferred to pCSV3 to produce plasmid pCSL68. The final construct bears a sequence of 1025 bp that comprises the *hetR* promoter and 27 bp of the 5’ end of the *hetR* ORF linked to the *gfp-mut2* gene by a sequence encoding four glycines. Plasmid pCSL68 was transferred to *Anabaena* sp. by conjugation, which was performed as described [52]. Clones resistant to streptomycin (Sm) and spectinomycin dihydrochloride pentahydrate (Sp) were selected and their genomic structure was tested by PCR. Periodically-performed PCR analysis indicated that the strains were stable, not showing wild-type chromosomes, in ammonium-containing medium supplemented with antibiotics. The *Anabaena* strains used as recipients in the conjugations were the wild-type PCC 7120, producing strain CSL64; CSVT20 (*patS* deletion mutant [33]), producing strain CSL65; CSL7 (*hetN* deletion mutant [27]), producing strain CSL66; CSSC2 (*hetR* deletion mutant[34]), producing strain CSL86; CSVM34 (*sepJ* deletion mutant [19]), producing strain CSVM10; and CSVT22 (*fraC fraD* deletion mutant [20]), producing strain CSVM11.

### Experiments

For experiments carried out under steady-state nitrogen-rich conditions, cells were taken from bubbled batch cultures in BG11_0_ medium with ammonium at 8–10 mM NH_4_Cl and, respectively, 16–20 mM TES-NaOH buffer (pH 7.5), and 10 mM NaHCO_3_ were supplemented with 1% CO_2_ in air. When required, antibiotics, streptomycin sulfate and spectinomycin dihydrochloride pentahydrate, were added to the media at final concentrations of 2 μg mL^-1^ for liquid and 5 μgmL^-1^ for solid media. Cultures were grown for 48 to 72 hrs in this medium as reported previously [27]. A sufficient concentration of ammonium (2–5 mM as determined with the Nessler reagent [53]) remained up to the time of sampling. For experimental runs performed under nitrogen deprivation, batch cultures grown with ammonium were harvested, washed with BG11_0_ medium and incubated in this medium with bicarbonate and CO_2_ as above. No antibiotics were added to the cultures incubated without combined nitrogen.

Samples taken from the cultures were set atop a thin slice of solidified medium in order to visualize the cells of the filaments in the same plane. The filaments were visualized using an immersion objective CX PL APO lambda blue 63.0x1.40 OIL UV objective in Leica SP5 microscope. Samples were excited at 488 nm using an argon laser. Fluorescence was monitored in the range 500–540 nm (GFP imaging) and 630–700 nm (cyanobacterial autofluorescence).

**Image segmentation**. All image processing and data analysis were performed using MATLAB (MathWorks). Cell recognition was performed on fluorescent images of cells using a program developed in our laboratory. In short, the program applies an adaptive threshold on the fluorescent images and check for the size of each cell, if the cell is bigger than a typical sized cell, the program increase the threshold for this cell. Using the adaptive threshold enable cells to be segmented correctly even with large variations in fluorescent intensity within each frame. The program’s output was checked manually in all experiments using bright-field images of the same frames, and corrected for errors in recognition. After segmentation, cells were manually separated and ordered into filaments. The average fluorescent from each cell was then extracted into Origin for statistical analysis.

**Data corpus:** Each experimental run for each strain consisted of 1350±350 (mean±std) cells, comprising 104±35 (mean±std) filaments. The minimal filament length analyzed was of 8 cells or longer.

**Statistical analysis.** Statistical comparisons between histograms of fluorescence were carried out using the Earth Mover’s Distance algorithm [39]. For one-dimensional histograms {*x*_*i*_}, {*y*_*i*_} of n bins, the Earth Mover’s Distance *EMD(X*,*Y)* is given by:

$$EMD\left(X,Y\right)=\overset{n}{\underset{i=1}{\sum }}\left|CDX\left(i\right)-CDY\left(i\right)\right|$$ (2)

where *CDX(i)* and *CDY(i)* are the respective cumulative distributions:

$$CDX\left(i\right)=\sum_{j=1}^{i}x_{i}$$

and similarly for *CDY(i)*. The EMD value corresponds to the total work required to move counts between columns in order to transform histogram *X* into *Y*, under the optimal scheme for doing so, and therefore uses all columns of the histograms homogeneously. In contrast, the more conventional Kolmogorov-Smirnov test, is based on a statistic *D*_*KS*_ that involves only the maximal difference between the bins of the cumulative distributions:

*D*_*KS*_ = sup_*i*_|*CDX*(*i*) – *CDY*(*i*)| The EMD is therefore a more intuitive measure to compare two histograms.

Comparisons between non-normalized cluster size histograms were carried out by computing the statistic:

$$X^{2}=\frac{1}{MN}\overset{b}{\underset{i=1}{\sum }}\frac{{\left(Mn_{i}+Nm_{i}\right)}^{2}}{n_{i}+m_{i}}$$ (3)

Here *b* is the number of bins, and N and M are the total number of events in each of the two histograms. *X* ^*2*^ has approximately a $\chi_{b-1}^{2}$ distribution [41]

## Supporting Information

S1 Fig Compensation for the negative bias in the spatial autocorrelation functions of fluorescence along filaments. The spatial autocorrelation function *g(n)* is a negatively-biased statistical estimator when the population mean is unknown and needs to be estimated from sampled data [40]. Calculation of *g(n)* directly from its definition (Eq. 1) and averaging over all filaments in an individual run clearly exhibits this bias for *n* ≥ 1, which decreases systematically with increasing distance n (solid lines in the above panels for the wild-type (WT), Δ*patS* andΔ*sepJ* strains). To compensate for the bias, we randomized cell positions within each filament, calculated the corresponding autocorrelation function and repeated this procedure ten times. The mean autocorrelation functions of filaments of permuted cells are shown in the above panels, for the respective strains with dashed lines. The autocorrelation functions shown in the main text (Fig. 3B and 3C), represent the compensation of *g(n)* with the permuted estimation of the bias for *n* ≥ 1.
(EPS)

S2 FigExpression of *hetR-gfp* in a strain lacking *hetR* positive auto-regulation (Δ*hetR*) does not change significantly following nitrogen deprivation.Normalized histograms of cell fluorescence of *hetR-gfp* under nitrogen-replete conditions (0 hrs), and at two times, 7 and 24 hours after nitrogen deprivation. The histograms are comprised of typically ∼1500 cells. The value of the protein noise$\sigma_{p}^{2}/\mu_{p}^{2}$, where *σ*_*p*_ is the standard deviation and *μ*_*p*_ the mean of *hetR-gfp* fluorescence distributions over the filaments is shown in each histogram. Noise errors were evaluated from 1000 bootstrap samples.(EPS)
